# Demographic and Socioeconomic Disparities in Telemedicine Use Among Individuals With Type 2 Diabetes in Primary Care: Systematic Review and Meta-Analysis

**DOI:** 10.2196/73113

**Published:** 2025-09-09

**Authors:** Nawwarah Alfarwan, Maria Panagioti, Alexander Hodkinson, Lamiece Hassan, Salwa S Zghebi, Evangelos Kontopantelis

**Affiliations:** 1 Division of Informatics, Imaging and Data Sciences, University of Manchester, Oxford Rd, Manchester, M13 9PL, United Kingdom, 44 7534135812; 2 Health Informatics Department, College of Health Sciences, Saudi Electronic University, Riyadh, Saudi Arabia; 3 Division of Population Health, Health Services Research and Primary Care, University of Manchester, Manchester, United Kingdom; 4 NIHR Greater Manchester Patient Safety Research Collaborations, University of Manchester, Manchester, United Kingdom; 5 National Institute for Health Research (NIHR) School for Primary Care Research, University of Manchester, Manchester, United Kingdom; 6 Division of Psychology and Mental Health, Manchester Academic Health Science Centre, University of Manchester, Manchester, United Kingdom

**Keywords:** type 2 diabetes, telemedicine, primary care, demographic and socioeconomic disparities, telehealth

## Abstract

**Background:**

Telemedicine has revolutionized the management of type 2 diabetes mellitus (T2DM) in primary care by improving access to health care services and enhancing health outcomes. Despite these advancements, it remains unclear whether telemedicine has reduced access inequalities among different demographic and socioeconomic groups.

**Objective:**

This study aimed to investigate the most important demographic and socioeconomic factors associated with telemedicine use among individuals with T2DM in primary care.

**Methods:**

We conducted a systematic review and meta-analysis. Databases including MEDLINE, Embase, PsycINFO, Google Scholar, Scopus, and CINAHL were searched from inception to December 2023. The reference lists of eligible studies and other relevant systematic reviews were also searched. We included observational and cohort studies that assessed the effects of telemedicine interventions on individuals with T2DM in primary care. The core outcomes were the factors associated with telemedicine use, reported as adjusted odds ratios and 95% CIs for each factor, using a random-effects model. Heterogeneity was quantified using the *I*² statistic, and publication bias was assessed. The protocol for this review was registered with PROSPERO (CRD42024550410).

**Results:**

Of the 3006 records identified, 16 studies involving 71,336 patients were included in the meta-analysis. Female patients had higher odds of using telemedicine than males (pooled adjusted odds ratio [OR] 1.05, 95% CI 1.02-1.09). Older patients were significantly less likely to use telemedicine than younger patients (pooled OR 0.979, 95% CI 0.98-0.98). Compared with White patients, Black patients were less likely to use telemedicine (pooled OR 0.55, 95% CI 0.32-0.94), while no statistically significant differences were observed for Hispanic (pooled OR 1.075, 95% CI 0.36-3.24) or Asian participants (pooled OR 0.56, 95% CI 0.29-1.06). Patients with higher education levels had greater odds of using telemedicine than those with lower education levels (pooled OR 1.681, 95% CI 1.48-1.91).

**Conclusions:**

This systematic review and meta-analysis provide evidence of significant disparities in telemedicine use among men, older adults, Black individuals, and those with lower levels of education who have T2DM in primary care. Given that these groups are among the most vulnerable to T2DM, these disparities highlight the critical need for strategic interventions and robust policies that ensure telemedicine fosters equitable access to health care while preventing further exacerbation of existing health inequalities.

## Introduction

Every 10 seconds, a person dies from diabetes-related complications worldwide [1]. In 2022, the global prevalence of diabetes reached an alarming 828 million, a dramatic increase from 198 million in 1990 [2]. Type 2 diabetes mellitus (T2DM) is a significant contributor to this rise, with an estimated 462 million people affected, making up 6.28% of the world’s population [3]. This escalating trend disproportionately impacts already vulnerable populations, particularly racial and ethnic minorities, who face compounded challenges in accessing essential health care services [4,5]. In the United Kingdom, the prevalence of T2DM among Asian and Black ethnic groups is 2- to 4-fold higher than that of White and other ethnic groups [6]. This troubling increase highlights the urgent need to improve health care services for underserved populations with T2DM.

In response to the growing diabetes prevalence and the increasing demand for accessible health care services, telemedicine has become an important component of the health care system [7,8]. Telemedicine refers to the delivery of health care services over distances using advanced technologies. It integrates a variety of approaches, such as online consultations, wearable devices, and mobile health apps, allowing health care providers to deliver equitable, affordable, and high-quality care remotely for individuals with T2DM [9]. Numerous studies emphasize the effectiveness of telemedicine, showing that it is not only as effective as traditional care but, in some cases, even more effective in improving health outcomes, optimizing diabetes management, and enhancing patient engagement [10,11].

Despite the transformative promise of telemedicine in revolutionizing diabetes care, substantial challenges continue to impede its effective management of T2DM, particularly among underserved communities [4,12]. Preliminary evidence highlights persistent disparities, with racial and ethnic minorities, especially Black individuals, being significantly less likely to use telemedicine compared with their White counterparts [13]. Furthermore, uninsured individuals or those with limited health coverage demonstrate markedly lower telemedicine adoption rates compared with those with private or public insurance [14]. These disparities are exacerbated by multifaceted barriers, including inadequate digital literacy, poor health literacy, restricted access to digital devices, and language proficiency limitations, which collectively hinder equitable telemedicine adoption in vulnerable populations [15,16].

Understanding the distinct characteristics of individuals with T2DM is essential for designing interventions that address disparities in telemedicine access and improve health care delivery for diabetic patients. A key challenge is understanding how various demographic and socioeconomic factors influence the adoption and use of telemedicine among individuals with T2DM in primary care. This study aims to bridge these gaps by conducting a comprehensive systematic review and meta-analysis of existing literature, exploring the relationships between demographic and socioeconomic factors and their impact on telemedicine adoption and use for T2DM care.

## Methods

### Overview

A systematic review was conducted and reported in accordance with the PRISMA (Preferred Reporting Items for Systematic Reviews and Meta-Analyses) statement [17]. The protocol for this review was registered with PROSPERO (CRD42024550410).

### Selection Criteria

The eligibility criteria for study inclusion were developed using the Population, Intervention, Comparison, Outcome, and Study (PICOS) framework (Multimedia Appendix 1).

### Information Sources

Databases searched included MEDLINE, Embase, PsycINFO, Google Scholar, Scopus, and CINAHL from inception through 31 December 2023. In addition, the bibliographies of relevant systematic reviews were reviewed to identify further publications, and content experts, as well as prolific authors in the field, were contacted.

### Search Strategy

A search strategy using Medical Subject Headings terms was designed to target 4 key domains: telehealth or telemedicine, primary care, adoption, and use. Pilot searches were undertaken for each domain and combined concepts to ensure the strategy’s effectiveness. The search strategy included a combination of Medical Subject Headings terms and free-text keywords. The full MEDLINE (Ovid) search strategy used to identify studies is provided in Multimedia Appendix 2. There were no restrictions on language, and the reference lists of all included studies were also screened for additional literature. Furthermore, we contacted the library to obtain full-text papers not found through database searches and reached out to experts and prolific authors in the field for additional studies.

### Study Selection

Search results were imported into the EndNote (version 14; Clarivate Analytics) reference management software, where duplicates were removed automatically and double-checked manually. Titles and abstracts of all identified records were independently assessed by authors (NA and MP). The studies meeting all eligibility criteria were retained for independent full-text assessment against the selection criteria by the first reviewer (NA) and confirmed by 2 other reviewers (EK and MP). Any discrepancies between reviewers were resolved through discussion.

### Data Extraction

A standardized form was developed by NA and refined by EK to guide the extraction of study characteristics and outcomes. Data were extracted on demographic and socioeconomic factors associated with telemedicine use, including gender, age, ethnicity, education level, and insurance status, along with their reported adjusted odds ratios (ORs) and 95% CIs. The extraction was conducted by one reviewer (NA) and checked by a second reviewer (EK). Any disagreements were resolved through discussion or, if needed, by a third reviewer (MP). The inter-rater reliability was excellent (κ=0.90).

### Risk of Bias Assessment

The quality appraisal of the studies to assess the risks of bias was carried out using the ROBINS-I (Risk of Bias In Non-randomized Studies of Interventions) tool, which is recommended by the Cochrane Scientific Committee for nonrandomized studies of interventions [18]. The ROBINS-I assesses the risk of bias in 7 domains, which include missing data, classification of interventions, selection of participants, deviations from intended interventions, measurement of the outcome, and selection of the reported result. The first reviewer (NA) completed the risk of bias assessment on all selected studies, which was confirmed by 4 other reviewers (EK, MP, LH, and SSZ).

### Data Synthesis

A meta-analysis was performed using random-effects models on the extracted data from the selected studies. A pooled adjusted OR for all outcomes was calculated. The study outcomes were pooled using a nonparametric bootstrap of the DerSimonian-Laird random-effects method (1000 iterations) [19] implemented in the Stata package (StataCorp) mean [20]. A random-effects model was preferred because it is a more conservative approach compared with a fixed-effect model [21], especially when heterogeneity is expected.

The assumption of homogeneity of true effect sizes was assessed using the Cochran Q test, and the degree of inconsistency across studies (*I*²) was calculated [22]. *I*² describes the percentage of total variation across studies that is due to heterogeneity rather than sampling error, and it ranges from 0% (no inconsistency) to 100% (high heterogeneity), with values of 0%‐40% suggesting low heterogeneity, 30%‐60% moderate heterogeneity, 50%‐90% substantial heterogeneity, and 75%‐100% considerable heterogeneity [23].

For each meta-analysis with 10 or more studies, funnel plots, the Begg test, and the Egger test were used to examine publication bias [24]. The trim-and-fill method was used as a sensitivity analysis to assess possible small-study publication bias.

### Ethical Considerations

Ethical approval and patient consent were not required, as they had already been obtained from the primary authors during their trial period. An ethical waiver was provided and acknowledged by our university’s institutional review board.

## Results

### Overview

**Figure 1.**
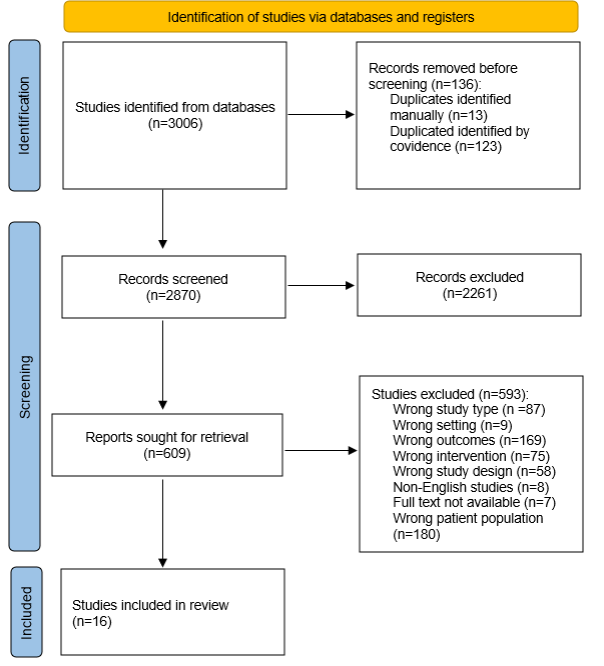
Flowchart of the inclusion of studies in the review.

The research initially yielded 3006 citations. After removing duplicates and reviewing the titles and abstracts, 2261 studies were excluded. Of the remaining 609 studies, 593 were excluded after reviewing the full texts (Figure 1). A total of 16 studies were included in the review [25-40].

### Characteristics of Included Studies

This review includes a pooled sample of 71,336 patients, with individual sample sizes ranging from 115 to 38,399 (median 1133, IQR 551-2214). Most studies were conducted in the United States (n=10, 62% studies), followed by the Netherlands (n=4, 25%) and the United Kingdom (n=2, 13%). Most studies had a cross-sectional design (n=10, 63%), while 6 (38%) studies used a cohort design. Most studies focused on patients with T2DM (n=6, 38%), 5 (31%) studies included populations where more than 50% had T2DM, and 5 (31%) studies did not specify the type of diabetes. Most studies were conducted in primary care settings (n=14, 88%), with 2 studies conducted in both primary and secondary care settings. Among the studies reviewed, 13 used asynchronous interventions (eg, web-based portals, secure messaging, text messages, and store-and-forward images), 2 used synchronous interventions (eg, real-time video consultations and telephone-based counseling), and 1 combined both types of interventions. The characteristics of all studies included in this review are presented in Multimedia Appendix 3.

### Risk of Bias Results

The ROBINS-I tool was used to assess the risk of bias in the included studies [12]. Among these, 5 studies [26,31,34,38,40] were found to have a low risk of bias, while 8 studies [25,28-30,32,33,36,39] exhibited a medium risk of bias. A critical risk of bias due to missing data was identified in 3 studies [27,35,37]. The risk of bias assessment for all studies is presented in Multimedia Appendix 4.

### Demographic Characteristics

#### Gender

**Figure 2.**
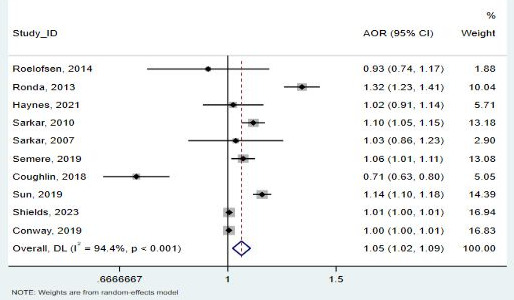
Forest plot for the association between gender and telemedicine use [25,26,29,30,32,33,36,37,39,40]. AOR: adjusted odds ratio.

**Figure 3.**
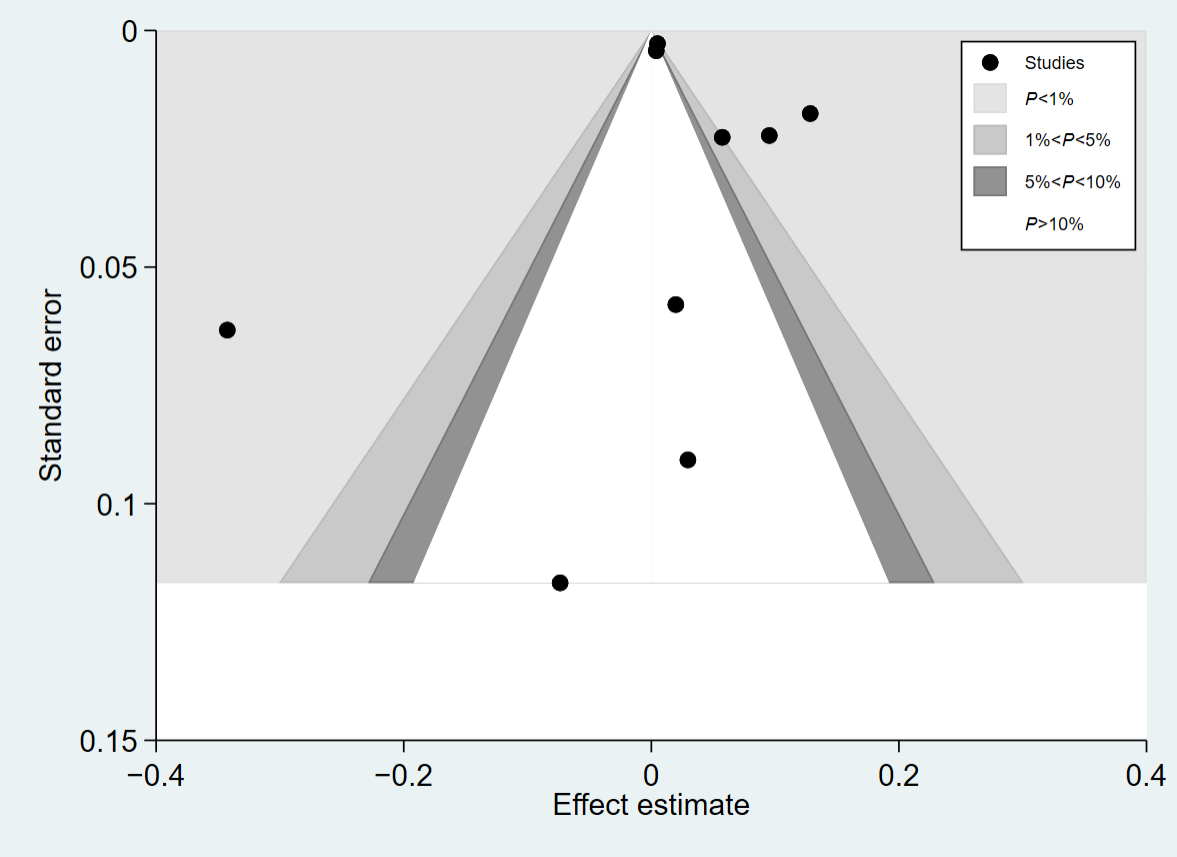
Funnel plot for the association between gender and telemedicine use.

Gender was evaluated in 10 studies (68,355 patients) [25,26,29,30,32,33,36,37,39,40]. This meta-analysis found that the use of telemedicine intervention was significantly higher among women than men (pooled OR 1.053, 95% CI 1.02-1.09; *I*²=94.4%; Figure 2). No publication bias regarding gender and the use of telemedicine was observed in the funnel plot (Figure 3). The Egger test also revealed no statistical significance for publication bias (*P*=.24).

#### Age

**Figure 4.**
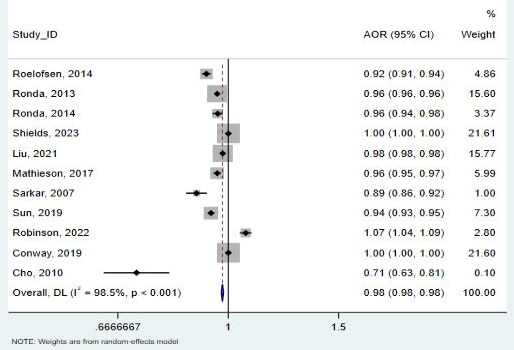
Forest plot for the association between age and telemedicine use [25-30,34,36,38,40,41]. AOR: adjusted odds ratio.

**Figure 5.**
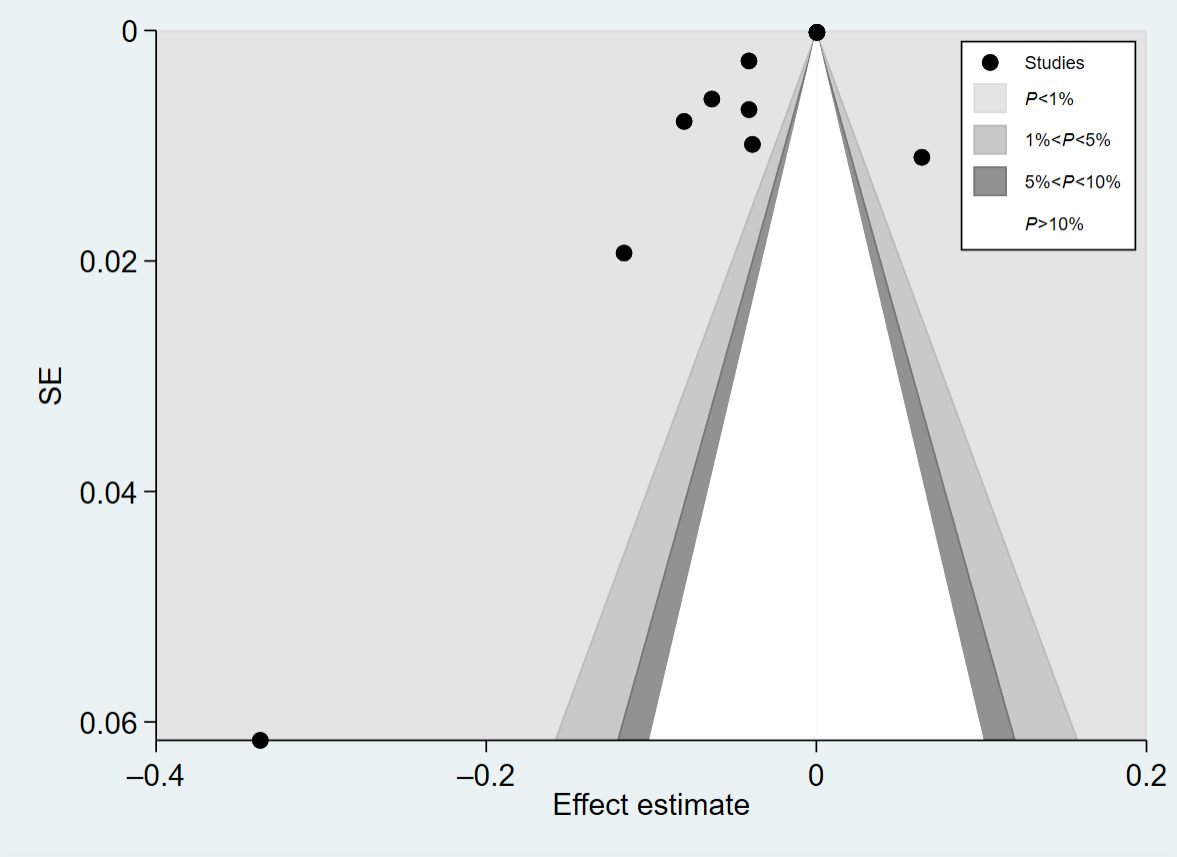
Funnel plot for the association between age and telemedicine use.

**Figure 6.**
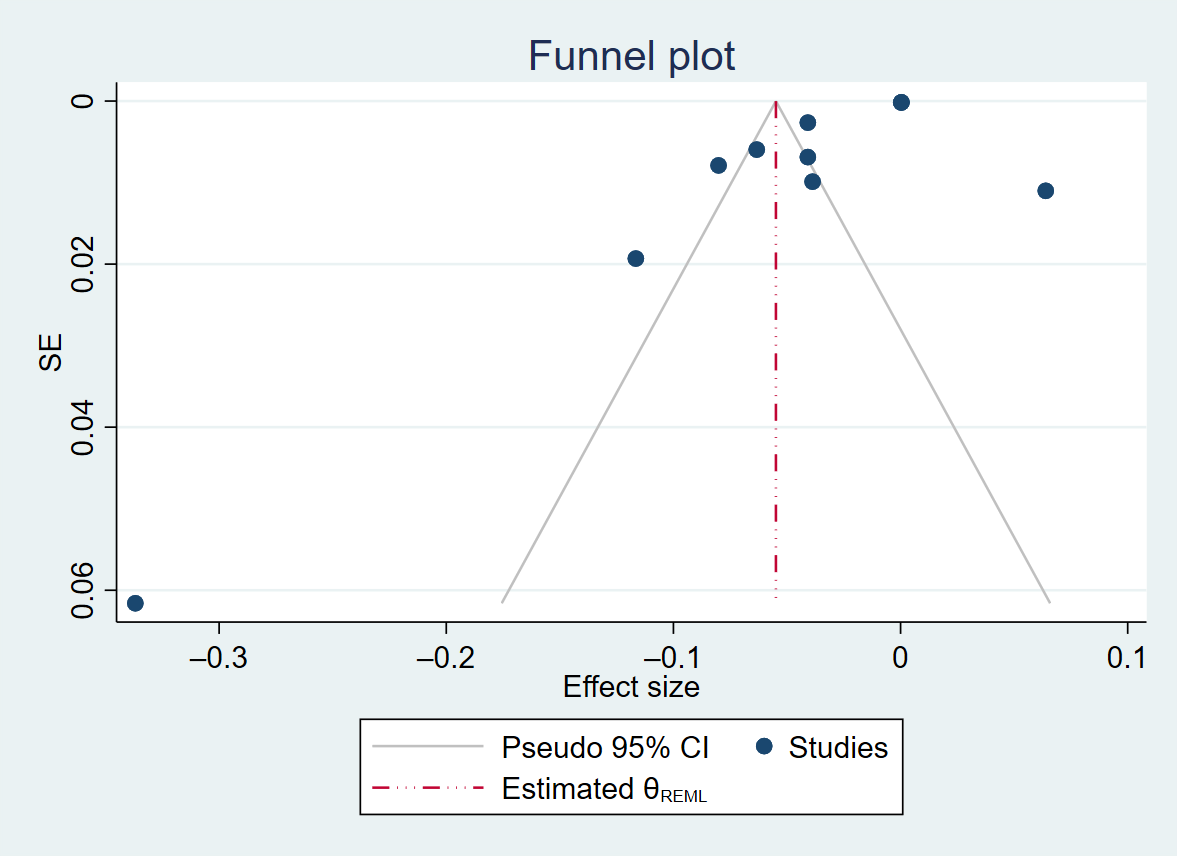
Trim-and-fill funnel plot for the association between age and telemedicine use.

Age was evaluated in 10 studies (47,927 patients) [25-30,33,34,36-41], 11 of which were included in the meta-analysis (Figure 4). It was not possible to pool data from 3 studies [33,37,39] due to age being reported categorically rather than as means and SDs. In this meta-analysis, “older” participants are defined as those whose age was 1 year above the mean age reported in each study. The results indicate that older adults showed lower use of telemedicine than younger adults (pooled OR 0.98, 95% CI 0.98-0.983; *I*²= 98.5%). The funnel plot showed little asymmetrical distribution (Figure 5), and the Egger test indicated potential small-study effects for publication bias (*P*<.001). However, the trim-and-fill analysis showed no substantial evidence of publication bias (Figure 6). The observed pooled effect size was 0.95 (95% CI 0.91-0.99), and no studies were imputed, indicating that publication bias is unlikely to meaningfully affect the conclusions of this meta-analysis.

#### Ethnicity

A total of 9 studies examined racial disparities in the use of telemedicine among individuals with T2DM: 5 among Black participants [31-33,36,39], 3 studies among Hispanic participants [32,36,39], 3 among Asian or Pacific Islander participants [31-33], and 8 among other or unspecified racial groups [29-33,37,39,40].

##### Black Participants

**Figure 7.**
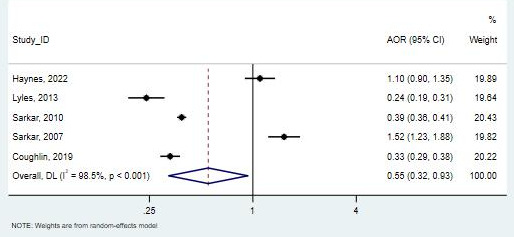
Forest plot for the association between Black race and telemedicine use [31-33,36,39]. AOR: adjusted odds ratio.

Of the 5 studies that included Black participants (13,717 patients), 3 studies observed a lower likelihood of telemedicine use compared with White participants [31,32,39], although 1 study was not statistically significant [33]. One study observed that Black participants had 52% higher odds of using telemedicine than White participants; however, the CI was wide, and this result could not be considered statistically significant [36]. In this meta-analysis, the overall OR of 0.55 (95% CI 0.32-0.94; *I*²=98.5%) shows that Black participants have significantly lower odds of using telemedicine compared with White participants. This result is statistically significant and highlights a disparity in telemedicine use among Black participants (Figure 7).

##### Hispanic Participants

**Figure 8.**
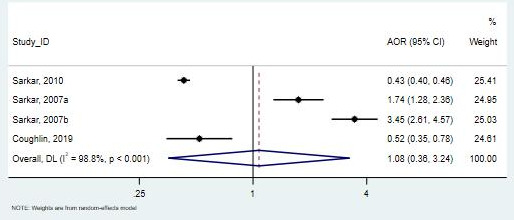
Forest plot for the association between Hispanic ethnicity and telemedicine use [32,36,39]. AOR: adjusted odds ratio.

Of the studies examining Hispanic participants (11,863 patients) [32,36,39], 2 found that Hispanic participants were less likely to use telemedicine compared with White participants, with both results being statistically significant [32,39]. In contrast, 1 study, which included both English- and Spanish-speaking Hispanic participants, found a higher likelihood of telemedicine use among Hispanic participants [36]. These 3 studies provided highly heterogeneous evidence when comparing Hispanic to White participants, with the meta-analysis result being nonsignificant (pooled OR 1.075, 95% CI 0.36-3.24; *I*²=98.8%; Figure 8).

##### Asian Participants

**Figure 9.**
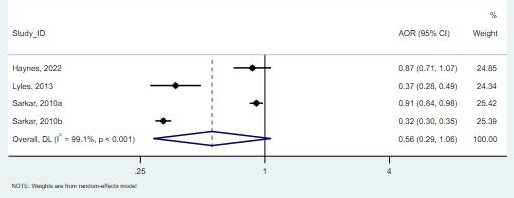
Forest plot for the association between Asian ethnicity and telemedicine use [31-33]. AOR: adjusted odds ratio.

Of the studies examining Asian participants (11,520 patients) [31-33], all 3 studies observed a lower likelihood of using telemedicine compared with White participants. One study reported an OR of 0.87 (95% CI 0.57-1.32), suggesting a 13% lower likelihood of using telemedicine among Asian participants compared with White participants, but this result was not statistically significant [33]. In contrast, another study found a 63% decrease in the likelihood of using telemedicine, and this result was statistically significant. The third study examined 2 groups: Asian and Filipino participants. For Asian participants, the OR was 0.9 (95% CI 0.77-1.05), indicating a 10% lower likelihood of using telemedicine, but this result was not statistically significant. For Filipino participants, the OR was 0.323 (95% CI 0.28-0.39), suggesting a 67.7% lower likelihood of using telemedicine compared with White participants, and this result was statistically significant. In the meta-analysis, the overall OR of 0.56 (95% CI 0.29-1.06; *I*²=99.1%) suggests that Asian participants have lower odds of using telemedicine compared with White participants. However, the result is not statistically significant (Figure 9).

##### Other Races or Unspecified Racial Groups

**Figure 10.**
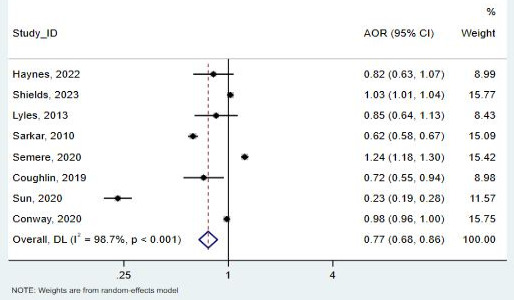
Forest plot for the association between other or unspecified racial groups and telemedicine use [29-33,37,39,40]. AOR: adjusted odds ratio.

Of the 8 studies (66,923 patients) that included other races or unspecified racial groups [29-33,37,39,40], 2 studies observed a statistically significant increase in telemedicine use among these groups compared with White participants [29,37]. In contrast, 4 studies reported a statistically significant decrease in telemedicine use among these groups compared with White participants [30,32,39,40]. In addition, 2 studies reported lower odds of using telemedicine, but the result was not statistically significant [31,33]. In this meta-analysis, the overall OR of 0.765 (95% CI 0.68-0.87; *I*²=98.7%) shows that other races or unspecified racial groups have significantly lower odds of using telemedicine compared with White participants. This result is statistically significant and highlights a disparity in telemedicine use among these groups (Figure 10).

### Socioeconomic Factors

#### Education

Five studies (59,609 patients) were included in this meta-analysis to assess the association between education and the use of telemedicine [26,28,32,37,40]. The ORs from these studies suggest that individuals with higher education are more likely to use telemedicine than those with lower education, with statistically significant findings in all studies. There was substantial heterogeneity between studies (*I*^2^=88.5%; *P*<.001); therefore, caution is needed when interpreting the pooled effect estimate. The pooled OR was highest among individuals with higher education compared with those with lower education (pooled OR 1.681, 95% CI 1.48-1.91). This indicates that individuals with higher education have 68.1% higher odds of using telemedicine compared with those with lower education (Multimedia Appendix 5).

#### Residence

Two studies (39,691 patients) were included in this meta-analysis to assess the association between urban versus rural settings and the use of telemedicine [33,40]. The overall effect estimates for the use of telemedicine comparing urban versus rural populations suggested that there is a significant difference between rural and urban samples (OR 1.005, 95% CI 1.00-1.01), indicating that urban patients are significantly more likely to use telemedicine than rural patients (Multimedia Appendix 5). There was no observed heterogeneity among the included studies (*I*²=0%).

#### Insurance Status

The meta-analysis of 2 studies (39,691 patients) assessed the association between insurance type (public vs private) and the use of telemedicine. Both studies found that individuals with public insurance were significantly less likely to use telemedicine compared to those with private insurance [33,40]. The pooled effect estimate was 0.712 (95% CI 0.59-0.86), indicating that individuals with public insurance were about 28.8% less likely to use telemedicine compared to those with private insurance (Multimedia Appendix 5).

## Discussion

### Principal Findings

To our knowledge, this is the first meta-analysis examining the association between demographic, socioeconomic, and insurance status factors with the use of telemedicine among individuals with T2DM in primary care. Our analysis quantified the effects from 16 published studies, comprising a total of 71,336 participants, and revealed a significant association between gender and telemedicine usage, with women showing higher engagement. Interestingly, despite the rapid development and deployment of telemedicine, older adults reported lower usage rates compared to younger adults. In addition, we found a statistically significant reduction in telemedicine use among Black individuals compared with White individuals, while reductions among Hispanic and Asian individuals were not statistically significant. These disparities may reflect broader structural and social inequalities that influence access to telemedicine interventions. Furthermore, individuals with higher education levels, those residing in urban areas, and those with private insurance demonstrated greater use of telemedicine services.

### Comparison With Other Studies

This finding suggests that, in general, men are less likely to use telemedicine compared with their women counterparts. These results are consistent with recent studies [42-44]. This trend can be attributed to several factors. Women are more actively engaged with health care services, including preventive care, chronic condition management, and mental health support, not only for themselves but also for their children and older family members [45]. This increased responsibility makes telemedicine a valuable tool, offering a convenient and efficient way to manage health care. Therefore, telemedicine provides essential support, empowering women to prioritize their health and the well-being of themselves and their families [46,47].

In addition, our review found that older adults have lower rates of telemedicine use compared with younger adults in primary care settings. This aligns with existing research, which shows that older adults frequently encounter barriers such as limited access to telemedicine services, low digital literacy, and concerns about privacy and security, all of which contribute to slower adoption rates [48-50]. This is a significant finding, especially considering that the population of people aged 60 years and older from minority backgrounds is projected to increase by 80% since the 2011 census [51]. Addressing these barriers is crucial to ensuring that older adults can benefit from the convenience and accessibility of telemedicine.

Furthermore, this review highlights a significant disparity in telemedicine use, with adults of lower educational levels being approximately 70% less likely to use these services compared with their more educated counterparts. This disparity may be attributed to barriers such as limited access to technology, low digital and health literacy, and concerns about trust and privacy [52-54]. These challenges are particularly concerning given the higher prevalence of chronic conditions such as T2DM among lower socioeconomic groups, who often have lower educational levels. This underscores the urgent need to improve telemedicine accessibility to ensure equitable health care access for all [55,56]. Although many studies highlight the potential of telemedicine for racial and ethnic minorities [57-59], this meta-analysis showed a statistically significant 45% reduction in telemedicine use among Black individuals compared with White individuals. This disparity may be due to several barriers that affect telemedicine use, including limited access to digital infrastructure, mistrust in health care systems, language barriers, and inadequate insurance coverage or digital literacy support [60-62]. A combination of these factors can restrict not only access and availability but also the motivation of Black individuals to engage with telehealth services. Notably, evidence of heterogeneity was detected in the analysis concerning racial disparities, likely due to variations in the study population, data source, exposure definition, and analytical approaches. Nevertheless, the summarized evidence indicates that racial disparities in telemedicine use remain a significant public health challenge [12,63,64]. Therefore, it is crucial to identify factors that adversely affect telemedicine use among racial and ethnic minorities, with the digital divide being one of the key factors that may play a significant role [65]. The digital divide highlights that many people, especially those from minority backgrounds, lack affordable access to the necessary technology, such as computers, smartphones, and broadband access [66,67]. These findings highlight the importance of understanding the patterns of health-related technology use across racially and ethnically diverse populations to appropriately tailor interventions aimed at improving minority health and eliminating health disparities. By understanding these disparities and addressing the underlying factors, we can develop more inclusive and effective telemedicine interventions.

### Strengths and Limitations of the Study

This review has several notable strengths, including the inclusion of studies with relatively large sample sizes and methodological rigor, which significantly strengthen the overall analysis. However, several limitations must be acknowledged. First, only a few studies explicitly examined racial and ethnic disparities, revealing a significant research gap that highlights the urgent need for more inclusive and diverse research. Second, all included studies were conducted in developed countries, which limits the generalizability of the findings and excludes valuable research from low- and middle-income countries (LMICs). Third, although income, employment status, and living conditions are key indicators of socioeconomic status, these factors were either not reported or were reported inconsistently across studies, which prevented their inclusion in the meta-analysis. Fourth, we could not assess disparities in telemedicine access among privately insured individuals due to missing details on insurance providers and coverage. Fifth, although we did not apply a specific conceptual framework to guide our analysis, the absence of a guiding model is a potential limitation of this review. Previous studies examining disparities in telemedicine have drawn on frameworks such as Andersen’s Behavioral Model of Health Services Use and the Behavioral Model for Vulnerable Populations to interpret structural factors such as insurance coverage and socioeconomic status [68,69]. Finally, there was evidence of substantial heterogeneity among the studies, which complicates the ability to draw definitive conclusions.

### Policy Implications and Future Research

Our findings highlight the urgent need for policymakers, health care systems, and stakeholders to prioritize reducing disparities in telemedicine use among individuals with T2DM. This condition disproportionately affects historically underserved populations, exacerbating their vulnerability to severe complications. Addressing these disparities requires targeted strategies and the development of robust policies for key demographic groups, including men, older adults, ethnic minorities, individuals with limited education, those reliant on public insurance, and residents of rural or underserved communities. These populations face significant barriers to telemedicine adoption, including technological and socioeconomic challenges that exacerbate disparities in access and, consequently, health outcomes.

Furthermore, significant research gaps persist in understanding other critical factors driving these disparities, particularly the roles of the digital divide, health literacy, digital literacy, and language barriers. To tackle these issues effectively, comprehensive research is necessary to explain the structural and individual-level determinants that hinder telemedicine adoption and use. This research will provide the insights needed to design effective and robust interventions. These interventions must include co-design approaches involving both patients and health care providers. Patients bring essential insights into lived experiences and challenges, while providers can identify systemic barriers and contribute to designing pragmatic, scalable solutions.

To build a resilient telemedicine infrastructure, we need sustained support for health care organizations, adherence to complex legal and regulatory frameworks, equitable and consistent funding mechanisms, and culturally tailored community engagement. Such foundational measures will ensure that telemedicine is not only accessible but also equitable and sustainable. Furthermore, a multipronged strategy is needed, framed within the principles of established frameworks [70]. This includes addressing structural barriers, promoting digital equity, enhancing research efforts, and fostering collaboration between patients and providers [71]. These approaches align with the American Medical Association’s Return on Health Telehealth Framework, which emphasizes the importance of closing digital gaps and creating fair, inclusive telemedicine systems [72]. Investments in telemedicine infrastructure should include training programs for health care providers, improved access to digital technologies for underserved populations, and communication campaigns highlighting telemedicine’s benefits, such as reduced travel times and timely care access. Such measures are particularly critical for older adults and other high-risk groups [73]. Building trust in telemedicine systems and addressing fears around technology adoption within the scope of this framework are key to driving greater use and improving health outcomes [74].

There is an urgent need to address evidence gaps in LMICs, where socioeconomic and demographic inequalities are often underrepresented in telemedicine research [74]. Policy makers must prioritize investing in targeted research to better understand the barriers faced by underserved populations in these communities. This includes funding studies that capture the sociocultural, economic, and technological factors influencing telemedicine use. By supporting robust data collection, promoting cross-sector collaboration, and ensuring equitable distribution of resources, policymakers can bridge these critical gaps. These actions will enable the development of inclusive telemedicine solutions tailored to the unique needs of LMICs, ultimately improving health care equity and access for the most vulnerable populations.

This systematic review and meta-analysis is the first comprehensive synthesis to examine disparities in the use of telemedicine among individuals with T2DM in primary care. While it offers valuable insights into the field of telemedicine, additional investigation is needed to better understand the social, structural, and technological factors driving these disparities. It is also important to explore how demographic, socioeconomic, and ethnic characteristics influence both the quality of care and the effectiveness of telemedicine interventions on clinical outcomes. Furthermore, future studies should assess whether the design or algorithmic components of telemedicine technologies contribute to unequal access or diagnostic accuracy among vulnerable populations. Research should also examine how the place of residence may intersect with public health infrastructure to affect the quality and outcomes of telemedicine care. Finally, there is a critical need to develop evidence-based policies that inform the design and implementation of equitable telemedicine solutions to prevent health inequalities and ensure fair access for all individuals with T2DM.

### Conclusion

This systematic review and meta-analysis provides evidence of significant disparities in telemedicine use among men, older adults, Black individuals, and those with lower levels of education who have T2DM in primary care. Given that these groups are among the most vulnerable to T2DM, these disparities highlight the critical need for strategic interventions and robust policies that ensure telemedicine fosters equitable access to health care, while preventing further exacerbation of existing health inequalities. Furthermore, we strongly urge further studies in this area, as we anticipate telemedicine will remain a critical care modality for individuals with T2DM in the coming years. Policy makers and health care stakeholders must take proactive measures to mitigate disparities, enhance access, and ensure that telemedicine fulfills its potential to improve outcomes for all individuals with T2DM.

## Supplementary material

10.2196/73113 Multimedia Appendix 1 Eligibility criteria for study inclusion.

10.2196/73113 Multimedia Appendix 2 MEDLINE search strategy.

10.2196/73113 Multimedia Appendix 3 Characteristics of included studies.

10.2196/73113 Multimedia Appendix 4 Assessment of risk of bias.

10.2196/73113 Multimedia Appendix 5 Additional forest plots of meta-analyses examining demographic and socioeconomic factors associated with telemedicine use.

10.2196/73113 Checklist 1 MOOSE checklist.

10.2196/73113 Checklist 2 PRISMA 2020 checklist.

## Abbreviations

LMIC: low- and middle-income country
OR: odds ratio
PICOS: Population, Intervention, Comparison, Outcome, and Study
PRISMA: Preferred Reporting Items for Systematic Reviews and Meta-Analyses
ROBINS-I: Risk of Bias In Non-randomized Studies of Interventions
T2DM: type 2 diabetes mellitus
