# Supplementary material for: Demographic and Socioeconomic Disparities in Telemedicine Use Among Individuals With Type 2 Diabetes in Primary Care: Systematic Review and Meta-Analysis
Source: J Med Internet Res. 2025 Sep 9;27:e73113. doi: 10.2196/73113 (PMC12419803; doi:10.2196/73113)
Supplement: Multimedia Appendix 1 [file jmir-v27-e73113-s001.docx]

Appendix 1: Eligibility criteria for study inclusion

| Population | Studies involving adults 18 years or over with a diagnosis of T2DM. Studies  included individuals with type 1 diabetes mellitus, women with gestational diabetes mellitus, and people at risk for diabetes mellitus. Additionally, the population was limited to individuals with T2DM, and studies involving both type 1 and type 2 diabetes patients were excluded unless specific data for individuals with T2DM was reported. Furthermore, studies that involved other health conditions were excluded unless they reported data specifically for individuals with T2DM. |
| --- | --- |
| Intervention | The two main types of telemedicine interventions; synchronous telemedicine (videoconferences and phone calls) and/or asynchronous telemedicine (messages, emails and other store-and-forward techniques). mHealth applications and telemonitoring interventions were excluded. |
| Comparator | All studies that utilized either historic or concurrent control groups, as well as those that did not employ any control group. |
| Outcome | The primary aim of this study is to explore individuals’ experiences and perceptions of using telemedicine interventions to manage T2DM. |
| Study setting | Studies conducted in primary care or community care settings were included, while studies conducted in home health care settings were excluded. |
| Study type | 1. Qualitative methodology or mixed methods. 2. The types of studies to be excluded are quantitative studies and mixed-method studies with predominantly quantitative results. |

…
