# Supplementary material for: Demographic and Socioeconomic Disparities in Telemedicine Use Among Individuals With Type 2 Diabetes in Primary Care: Systematic Review and Meta-Analysis
Source: J Med Internet Res. 2025 Sep 9;27:e73113. doi: 10.2196/73113 (PMC12419803; doi:10.2196/73113)
Supplement: Multimedia Appendix 2 [file jmir-v27-e73113-s002.docx]

**Medline search strategy**

Diabetes Mellitus, Type 2/ or diabetes.mp. or Diabetes Mellitus/

(((type 2 or type ii or non-insulin or noninsulin or adult onset or slow onset or maturity onset) adj3 diabet*) or T2DM or dmt2 or dm2 or T2-DM or dmt2 or dm-2 or NIDDM or NID-DM or MODY).ti,ab.

(Telehealth or tele-health or telemedicine or Tele-medicine or telecare or tele-care or telehealthcare or tele-healthcare or teleconsultation or tele-consultation or "video consultation" or video-consultation or videoconference or video-conference or "video conferencing" or teleconference or tele-conference or telecommunication or tele-communication or telerehab* or tele-rehab* or telecoaching or tele-coaching or ehealth or e-health or "electronic health" or ecare or e-care or "electronic care" or "digital health" or etherapy or e-therapy or "electronic therapy").mp.

Web Browser/

Patient Portals/

Online Systems/

Internet/

Computer Systems/

Telemedicine/

Telenursing/

(automat* adj3 (phone* or telephone* or call* or system*)).mp.

Family Practice/

Primary Health Care/

Physicians, Family/

Community Health Services/

Community Health Nursing/

Community Mental Health Services/

Community Mental Health Centers/

family pract$.tw.

general practice$.tw.

community based.tw.

community care.tw.

family medicine.tw.

family physician$.tw.

primary care.tw.

(primary health care or primary healthcare).tw.

family doctor$.tw.

primary medical care.tw.

general physician$.tw.

primary care practitioner$.tw.

(community adj (health or healthcare or health care)).tw.

primary healthcare team$.tw.

primary health care team$.tw.

primary medical care team$.tw.

practice nurse$.tw.

practice manager$.tw.

(gpsi or gpwsi).tw.

(practitioner$ adj3 special interest$).tw.

(primary care or primary health care or general practice or family practice or family medicine).tw.

((adapt* or adopt* or appl* or chang* or execut* or implement* or incorporat* or innovat* or interpret* or operational* or practi#e? or practi#ing or transfer* or utilis* or utiliz* or uptake) adj5 (aid or aids or aiding or aided or assist* or bar or barred or barring or barrier* or block* or challeng* or constrain* or deter* or difficult* or discourag* or disincentive* or encourag* or encumber* or encumbranc* or enhanc* or facilitat* or help* or hinder* or hindrance* or impair* or impede* or impeding or impediment* or influen* or interfer* or motivat* or obstruct* or problem* or promot* or restrain* or restrict*)).tw.

(facilitator* or motivation* or benefit*).mp.

(barrier* or challenge*).mp.

or/1-2

or/3-11

or/40-42

or/12-39

43 and 44 and 45 and 46

..
