# Supplementary material for: Demographic and Socioeconomic Disparities in Telemedicine Use Among Individuals With Type 2 Diabetes in Primary Care: Systematic Review and Meta-Analysis
Source: J Med Internet Res. 2025 Sep 9;27:e73113. doi: 10.2196/73113 (PMC12419803; doi:10.2196/73113)
Supplement: Multimedia Appendix 5 [file jmir-v27-e73113-s005.docx]

**Multimedia Appendix 4.** Additional forest plots of meta-analyses examining demographic and socioeconomic factors associated with telemedicine use.
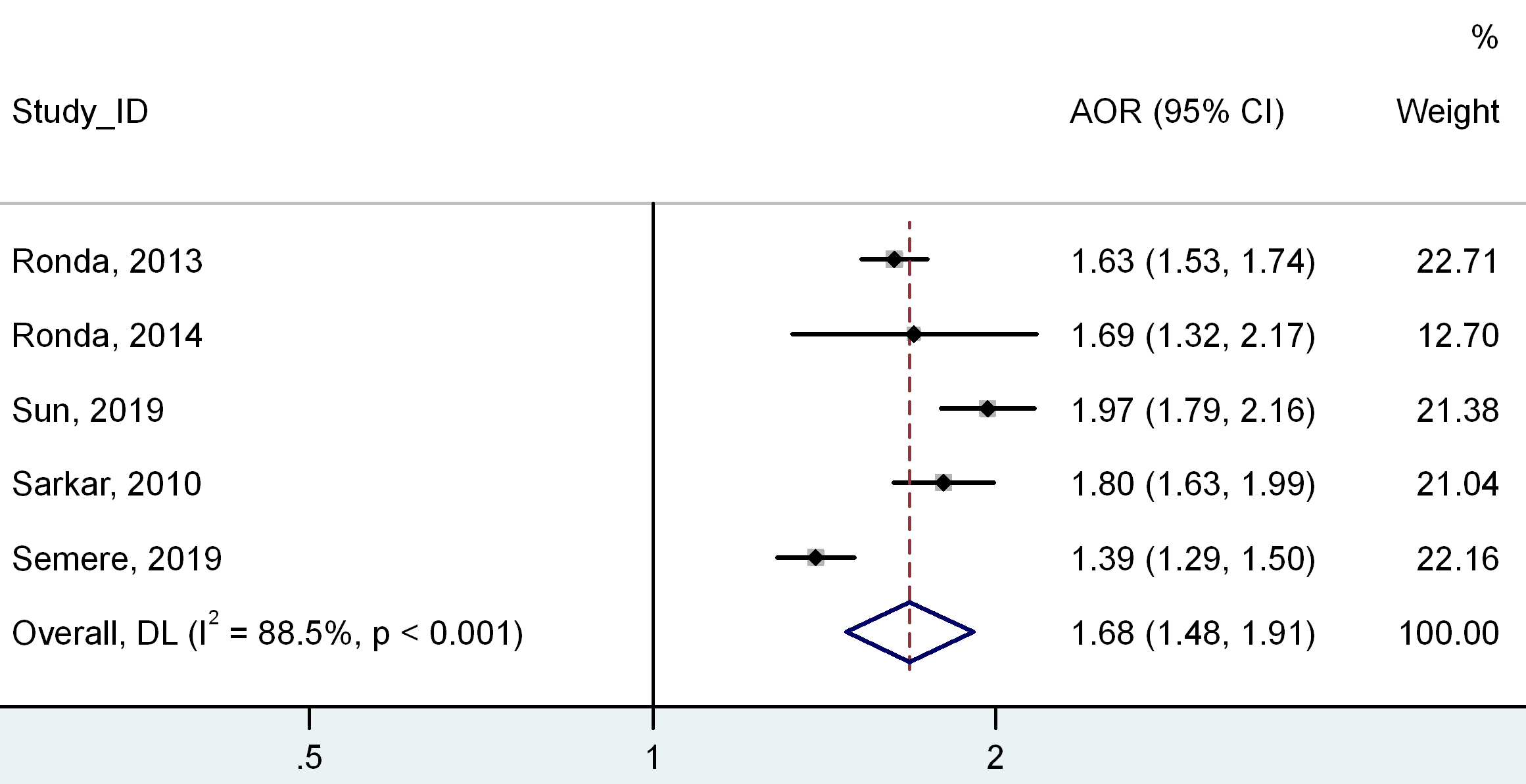


**Figure S1.** Forest plot for the association between education and the use of telemedicine in primary care.


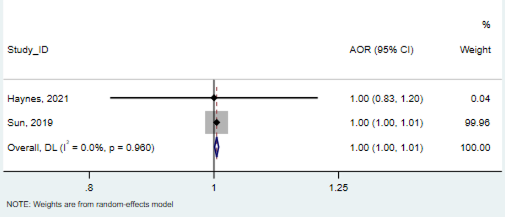


**Figure S2.** Forest plot for the association between residence and the use of telemedicine in primary care.


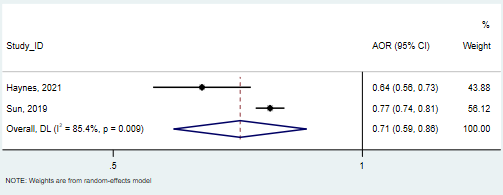


**Figure S3.** Forest plot for the association between insurance type and the use of telemedicine in primary care.

…
